# Factors associated with preferences for long-term care settings in old age: evidence from a population-based survey in Germany

**DOI:** 10.1186/s12913-017-2101-y

**Published:** 2017-02-21

**Authors:** André Hajek, Thomas Lehnert, Annemarie Wegener, Steffi G. Riedel-Heller, Hans-Helmut König

**Affiliations:** 1 Department of Health Economics and Health Services Research, University Medical Center Hamburg-Eppendorf, Hamburg, Germany; 2 Institute of Social Medicine, Occupational Health and Public Health, University of Leipzig, Leipzig, Germany

**Keywords:** Care setting, Need for care, Preferences, Long-term care preferences, Old age, Germany

## Abstract

**Background:**

Long-term care is one of the most pressing health policy issues in Germany. It is expected that the need for long-term care will increase markedly in the next decades due to demographic shifts. The purpose of this study was to investigate the factors associated with preferences for long-term care settings in old age individuals in Germany.

**Methods:**

Based on expert interviews and a systematic review, a questionnaire was developed to quantify long-term care preferences. Data were drawn from a population-based survey of the German population aged 65 and over in 2015 (*n* = 1006).

**Results:**

In multiple logistic regressions, preferences for home care were positively associated with providing care for family/friends [OR: 1.6 (1.0–2.5)], lower self-rated health [OR: 1.3 (1.0–1.6)], and no current need of care [OR: 5.5 (1.2–25.7)]. Preferences for care in relatives’ homes were positively associated with being male [OR: 2.0 (1.4–2.7)], living with partner or spouse [OR: 1.8 (1.3–2.4)], having children [OR: 1.6 (1.0–2.5)], private health insurance [OR: 1.6 (1.1–2.3)], providing care for family/friends [OR: 1.5 (1.1–2.0)], and higher self-rated health [OR: 1.2 (1.0–1.4)]. Preferences for care in assisted living were positively associated with need of care [OR: 1.9 (1.0–3.5)] and higher education [for example, University, OR: 3.5 (1.9–6.5)]. Preferences for care in nursing home/old age home were positively associated with being born in Germany [OR: 1.8 (1.0–3.1)] and lower self-rated health [OR: 1.2 (1.0–1.4)]. Preferences for care in a foreign country were positively associated with lower age [OR: 1.1 (1.0–1.2)] and being born abroad [OR: 5.5 (2.7–11.2)].

**Conclusions:**

Numerous variables used are sporadically significant, underlining the complex nature of long-term care preferences. A better understanding of factors associated with preferences for care settings might contribute to improving long-term care health services.

**Electronic supplementary material:**

The online version of this article (doi:10.1186/s12913-017-2101-y) contains supplementary material, which is available to authorized users.

## Background

It is projected that the number and proportion of individuals in old age will increase considerably in the future decades [1]. Because old age is associated with the need for long-term care, it is projected that the number of individuals in need for long-term care will increase substantially [2].

Most often, it is assumed that individuals prefer to life at home as long as possible in order to maintain, e.g., social ties or familiar surroundings. Individuals prefer care settings with a high degree of autonomy [3]. If long-term care is needed, home care is often provided informally by relatives or friends which matches the preferences expressed by care-recipients [3]. Preferences shift toward inpatient care when the need for care grows [4–7].

Generally, care provided in the community is less costly for the system of social security. Therefore, health policy in Germany, like in many other countries, aims at promoting care provided in the community (§ 3 Social Security Code XI). Nevertheless, informal and formal caregiving in the community is very time-consuming, especially when care-recipients are severely cognitively impaired [8]. Furthermore, it is most likely that (i) the geographical distance of family members, (ii) the employment rates of women and (iii) the proportion of elderly individuals living alone will increase in the next decades [9, 10]. Thus, provision of informal care will most likely become more challenging [11]. Moreover, recent longitudinal studies have found that informal caregiving is associated with various negative outcomes for the caregiver, such as increased depressive symptoms [12, 13]. Furthermore, informal caregiving can eventually result in abusive behavior against the care-recipient. On the other hand, studies have also shown that informal caregiving is associated with several positive outcomes for the caregivers including greater self-esteem or personal growth [14].

For policy-makers as well as for various other stakeholders such as nursing services or informal caregivers, it is important to know which factors are associated with preferences for care settings. This might help to reduce the gap between long-term care preferences and reality which in turn might help to increase satisfaction of individuals in need for care [15, 16].

Yet, little is known about preferences for the various long-term care settings in older individuals in Germany. In particular, studies are missing that examine the *various predictors* of preferences for *care settings comprehensively*. Furthermore, only a few studies investigated the predictors of preferences for long-term care abroad [17–19]. Thus, by using a large population-based sample of individuals aged 65 and over, the purpose of this study was to investigate which factors are associated with preferences for care settings (1. Home care; 2. Care in relatives’ homes; 3. Care in assisted living; 4. Care in nursing home/old age home; 5. Care in a foreign country) in old age individuals in Germany. We focus on individuals in old age because these individuals are at high risk of needing long-term care [20, 21], and it was shown that they are more knowledgeable regarding different aspects of long-term care [22].

## Methods

### Sample

In 2015, *n* = 1006 individuals aged 65 and above living in private households with conventional telephone connection were interviewed by phone [23, 24] (Computer Assisted Telephone Interview, CATI). Fieldwork was carried out by USUMA (Berlin)—a company specialized in market and social research. The interviews lasted for about 25 min. Individuals were randomly selected from all registered private telephone numbers (using the Guidelines for Telephone Surveys from the *ADM Arbeitskreis Deutscher Markt- und Sozialforschungsinstitute e.V.*), enabling representative sampling. Furthermore, the numbers were computer-generated, allowing for ex-directory households as well. In addition, repeat calls were conducted at different times on different days of the week until an answer was given (if the telephone was not answered at the tenth attempt, the number was dropped). From the gross sample (*n* = 2346), *n* = 1006 interviews were realized (42.9%). Main reasons for refusal were lack of time/lack of interest (12.1%) and refusal to take part in telephone surveys (26.5%).

The authors of this study did not have any physical contact with the participants. Furthermore, personally-identifying information from study participants were not collected and responses were anonymized prior to analysis. Based on expert interviews [3] and a systematic review of the literature, a questionnaire was constructed to quantify long-term care preferences.

### Dependent variables

Individuals were asked to report their preferences for care settings: “When care is needed, I would like to be cared for …” (from 1 = “totally agree” to 4 = “totally disagree”)

- at own home
- in relatives’ homes
- in assisted living
- in nursing home/old age home
- in a foreign country

A mid-category (indifferent in the choice question) was not included because we prefer respondents to make a definite choice. The five dependent variables were all dichotomized (0 = “totally disagree” and “rather disagree”; 1 = “totally agree” and “rather agree”) to indicate high preferences versus low preferences.

### Independent variables

As explanatory variables in this study, we used socioeconomic factors as follows: age in years, sex (women; men), living situation (living with partner or spouse; others (living alone; living with other family members; living with other individuals), region (West Germany; East Germany), education (without a vocational degree; apprenticeship, full-time vocational school; professional school or trade and technical school for vocational education; University, school of engineering), place of birth (born in Germany; born abroad), having children (yes; no), status of health insurance (statutory health insurance; private health insurance). In addition, it was assessed whether the respondent provided informal care for family or friends (yes; no).

Furthermore, the current need of care was quantified by recording the level of care according to the German long-term care insurance: In order to claim for benefits of the long-term care insurance, individuals must need daily a minimum of 90 min of assistance with basic (instrumental) activities of daily living. Depending on the extent of care required, recipients are categorised into 3 levels after an assessment by a nurse or a physician of the medical service of the German statutory health insurance system. Need of care was dichotomized (0 = no level of care; 1 = level 1 to 3).

Subjective health was measured by using self-rated health, ranging from 1 (“very bad”) to 5 (“very good”). Moreover, the involvement in the issue of need for care (“How much have you thought about the issue of ‘need for care’”) was assessed by using a 5-point Likert scale (from 1 = “very little” to 5 = “very much”).

### Statistical analysis

Bivariate comparisons between the two groups (high preferences; low preferences) were done using t-test and chi-square procedures, as appropriate. Multiple logistic regressions were used to examine the relationship between predictors and the five dichotomized outcome measures separately (own home, relatives’ home, assisted living, nursing home/old age home, foreign country). Thus, five multiple logistic regressions were performed. The level of significance was chosen at a *p*-value of less than .05. Statistical analyses were performed using Stata 14.0 (StataCorp, College Station, Texas).

## Results

### Sample characteristics and bivariate analysis

Table 1 gives an overview of sample characteristics. For example, most of the individuals were female (56.7%). Mean age was 75.7 years (±6.6 years, ranging from 65 to 96 years). Nearly half of the individuals lived alone in own household (46.2%). 38.0% of the individuals hold an apprenticeship degree/full-time vocational school. 83.6% of the individuals had at least one child. Mean self-rated health was 3.6 (±0.9, ranging from 1 to 5) and 6.0% of the individuals were in need of care (level 1 to 3). Furthermore, Table 1 displays *bivariate* associations between our outcome measures and independent variables.

**Table 1:** Bivariate associations between preferences for care settings and independent variables

	Home care			Care in relatives’ homes			Care in assisted living			Care in nursing home/old age home			Care in a foreign country		
	Low preferences (*n* = 127; 12.8%)	High preferences (*n* = 866; 87.2%)	*p*-value^a^	Low preferences (*n* = 668; 67.7%)	High preferences (*n* = 319; 32.3%)	*p*-value^a^	Low preferences (*n* = 426; 44.4%)	High preferences (*n* = 534; 55.6%)	*p*-value^a^	Low preferences (*n* = 659; 68.0%)	High preferences (*n* = 310; 32.0%)	*p*-value^a^	Low preferences (*n* = 929; 94.5%)	High Preferences (*n* = 54; 5.5%)	*p*-value^a^
Age: Mean (SD)	76.5 (7.5)	75.5 (6.4)	*p* = .11	75.8 (6.7)	75.4 (6.4)	0.43	**76.3 (6.7)**	**75.0 (6.5)**	***p*** **< .01**	75.4 (6.6)	75.8 (6.5)	*p* = .33	**75.9 (6.6)**	**72.1 (5.6)**	***p*** **< .001**
Sex: N			***p*** **< .01**			***p*** **< .001**			*p* = .83			*p* = .49			*p* < .10
Women	**85**	**475**		**411**	**147**		238	302		367	180		538	24	
Men	**42**	**391**		**257**	**172**		188	232		292	130		391	30	
Living situation: N									*p* = .37			*p* = .93			*p* = .98
Living with partner or spouse	**35**	**353**	***p*** **< .01**	**216**	**168**	***p*** **< .001**	161	217		257	120		363	21	
Others	**92**	**513**		**452**	**151**		265	317		402	190		566	33	
Region: N									*p* = .97			*p* = .29			*p* = .21
West Germany	94	648	*p* = .84	490	244	*p* = .29	317	398		485	238		243	10	
East Germany	33	218		178	75		109	136		174	72		686	44	
Education: N						*p* = .98			***p*** **< .001**			*p* < .10			***p*** **< .05**
Without a vocational degree	10	65	*p* = .83	52	23		**45**	**23**		44	25		**67**	**5**	
Apprenticeship, full-time vocational school;	52	319		254	117		**158**	**201**		236	132		**363**	**10**	
Professional school or trade and technical school for vocational education;	28	214		162	79		**106**	**127**		168	62		221	17	
University, Fachhochschule, school of engineering	37	262		200	95		**114**	**180**		208	88		274	21	
Place of birth: N			*p* = .37			***p*** **< .05**			*p* = .27			*p* = .33			***p*** **< .001**
Born in Germany	120	796		**624**	**285**		390	500		605	290		**866**	**41**	
Born abroad	7	67		**43**	**32**		34	33		52	19		**60**	**13**	
Having children: N			*p* = .42			***p*** **< .01**			*p* = .96			*p* = .53			*p* = .23
Yes	103	726		**545**	**283**		356	448		554	256		779	42	
No	24	139		**123**	**35**		69	86		104	54		149	12	
Status of health insurance: N						***p*** **< .01**			*p* = .96			*p* = .65			*p* = .22
Statutory health insurance	111	734	*p* = .50	**586**	**257**		360	453		558	265		793	43	
Private health insurance	16	128		**81**	**60**		63	80		99	43		132	11	
Provided care for family/friends: N						*p* = .97			*p* = .39			*p* = .92			*p* = .57
Yes	67	410	*p* = .22	346	154		215	285		341	161		444	28	
No	59	456		321	154		210	249		318	148		484	26	
Level of care: N			**p > .05**			*p* = .62			***p*** **< .05**			*p* = .41			*p* = .48
Yes	**2**	**58**		39	21		**33**	**23**		43	16		57	2	
No	**125**	**805**		629	295		**392**	**510**		614	293		870	51	
Self-rated health (from x = ‘very bad’ to y = ‘very good’): Mean (SD)	**3.8 (0.8)**	**3.6 (0.9)**	***p*** **< .05**	**3.6 (0.9)**	**3.7 (0.9)**	***p*** **< .05**	3.6 (1.0)	3.7 (0.9)	*p* = .19	3.7 (0.9)	3.6 (0.9)	*p* = .11	3.6 (0.9)	3.6 (1.1)	*p* = .60
Involvement in the issue of need for care (from x = ‘very little’ to y = ‘very much’): Mean (SD)	3.0 (1.5)	2.9 (1.4)	*p* = .45	2.9 (1.5)	2.8 (1.4)	*p* = .16	2.9 (1.5)	2.9 (1.4)	*p* = .63	2.9 (1.4)	2.9 (1.4)	*p* = .61	2.9 (1.4)	2.8 (1.6)	*p* = .58

^a^Comparisons between the two groups were done using t-test and chi-square procedures; Significant findings (*p* < .05) were highlighted (bold)

### Regression analysis

In *multiple* logistic regressions (Table 2), preferences for home care were positively associated with providing care for family/friends [OR: 1.6 (1.0–2.5)], lower self-rated health [OR: 1.3 (1.0–1.6)], and no need of care [OR: 5.5 (1.2–25.7)]. Preferences for care in relatives’ homes were positively associated with being male [OR: 2.0 (1.4–2.7)], living with partner or spouse [OR: 1.8 (1.3–2.4)], having children [OR: 1.6 (1.0–2.5)], private health insurance [OR: 1.6 (1.1–2.3)], providing care for family/friends [OR: 1.5 (1.1–2.0)], and higher self-rated health [OR: 1.2 (1.0–1.4)]. Preferences for care in assisted living were positively associated with need of care [OR: 1.9 (1.0–3.5)] and higher education [for example, University, OR: 3.5 (1.9–6.5)]. Preferences for care in nursing home/old age home were positively associated with being born in Germany [OR: 1.8 (1.0–3.1)] and lower self-rated health [OR: 1.2 (1.0–1.4)]. Preferences for care in a foreign country were positively associated with lower age [OR: 1.1 (1.0–1.2)] and being born abroad [OR: 5.5 (2.7–11.2)].

**Table 2:** Predictors of preferences for care settings. Results of logistic regressions (for each outcome measure: 0 = low preferences; 1 = high preferences)

	(1)	(2)	(3)	(4)	(5)
	Home care	Care in relatives’homes	Care in assisted living	Care in nursing home/old age home	Care in a foreign country
Age	0.977	1.005	0.981^+^	1.014	0.902^***^
	(0.948–1.007)	(0.983–1.027)	(0.961–1.001)	(0.992–1.036)	(0.854–0.951)
Sex (Ref.: Male)	0.698	0.506^***^	1.176	0.963	0.650
	(0.433–1.125)	(0.366–0.700)	(0.864–1.600)	(0.694–1.337)	(0.331–1.275)
Living situation (Ref.: Living with partner or spouse)	0.674^+^	0.559^***^	1.016	0.951	1.306
	(0.432–1.051)	(0.410–0.762)	(0.758–1.364)	(0.697–1.297)	(0.684–2.493)
West and East Germany (Ref.: East Germany)	1.161	0.981	1.025	1.316	1.479
	(0.690–1.956)	(0.677–1.422)	(0.720–1.459)	(0.897–1.931)	(0.617–3.549)
Apprenticeship, full-time vocational school (Ref.: Without a vocational degree)	0.992	0.806	2.984^***^	0.962	0.246^*^
	(0.476–2.065)	(0.459–1.414)	(1.688–5.275)	(0.552–1.675)	(0.0720–0.841)
Professional school or trade and technical school for vocational education	1.269	0.834	2.666^**^	0.681	0.822
	(0.574–2.808)	(0.460–1.511)	(1.465–4.850)	(0.374–1.240)	(0.255–2.647)
University, Fachhochschule, school of engineering	1.062	0.562^+^	3.494^***^	0.733	0.858
	(0.471–2.393)	(0.305–1.036)	(1.892–6.452)	(0.398–1.350)	(0.261–2.820)
German-born (Ref.: No)	0.678	0.703	1.205	1.782^*^	0.184^***^
	(0.291–1.575)	(0.433–1.144)	(0.732–1.983)	(1.014–3.129)	(0.0910–0.374)
Children (Ref.: No children)	1.096	1.610^*^	1.132	0.872	0.669
	(0.647–1.854)	(1.047–2.475)	(0.780–1.644)	(0.592–1.285)	(0.322–1.391)
Status of health insurance (Ref.: statutory health insurance)	1.211	1.566^*^	0.918	0.978	0.849
	(0.648–2.263)	(1.055–2.324)	(0.627–1.343)	(0.648–1.476)	(0.388–1.858)
Provided care for family/friends (Ref.: No)	1.600^*^	1.468^*^	0.915	1.181	0.812
	(1.043–2.454)	(1.086–1.985)	(0.689–1.217)	(0.874–1.596)	(0.437–1.509)
Level of care (Ref.: No)	0.189^*^	0.573^+^	1.900^*^	1.715^+^	1.899
	(0.0406–0.879)	(0.317–1.035)	(1.045–3.453)	(0.903–3.257)	(0.284–12.70)
Self-rated health (from 1 = ‘very bad’ to 5 = ‘very good’)	0.762^*^	1.192^*^	1.012	0.850^*^	0.810
	(0.601–0.968)	(1.013–1.402)	(0.869–1.180)	(0.723–1.000)	(0.584–1.125)
Involvement in the issue of need for care (from 1 = ‘very little’ to 5 = ‘very much’)	0.894	0.978	1.037	0.980	0.976
	(0.772–1.036)	(0.881–1.086)	(0.939–1.146)	(0.883–1.089)	(0.786–1.211)
Constant	6647^***^	1.375	0.330	0.0709^*^	1188^*^
	(75.97–581,578)	(0.110–17.22)	(0.0289–3.761)	(0.00541–0.930)	(2.403–586,850)
Observations	974	968	942	950	964
Pseudo R^2^	0.043	0.059	0.027	0.017	0.137

*Comments*: Odd ratios were reported. 95% confidence intervals in parentheses. ^***^ *p* < 0.001, ^**^ *p* < 0.01, ^*^ *p* < 0.05, ^+^ *p* < 0.10.

## Discussion

By using a large, population-based sample of individuals aged 65 and above in Germany, the aim of this study was to examine which factors are associated with preferences for long-term care settings. We found that preferences for home care were *positively* associated with providing care for family or friends. This is in accordance with a recent population-based study among individuals aged 45 and above in Germany [25] and might be explained by the fact that individuals who already provided informal care are familiar with home care, so that the situation can be understood. Moreover, feelings of reciprocity (giving with expectation of future reward) might be important. In addition, it might be explained by strong family networks.

Preferences for home care were significantly associated with sex, living situation, need of care and self-rated health. Furthermore, sex, living situation, place of birth, having children and self-rated health were significantly associated with preferences for care in relatives’ homes. In addition, preferences for care in assisted living were significantly associated with age, education and need of care. Moreover, preferences for care in nursing home/old age home were not significantly associated with included independent variables. Age, education and place of birth were significantly associated with preferences for care in a foreign country.

Furthermore, we found that preferences for care in relatives’ homes were positively associated with being male, and living with partner or spouse, which is also in line with previous findings [25, 26]. The former association might be explained by the fact that older women might prefer nursing home facilities when care needs are substantial [27]. Kasper et al. [27] have also shown that their husbands saw in-home family care as the best caregiving arrangement. However, there is also equivocal data suggesting that older men (aged 40–70) preferred care in paid/professional settings, whereas older women more often preferred kin/home care [28]. In total, we assume that the sense of being a burden to relatives might differ markedly between women and men. Nevertheless, only a few studies examined self-perceived feelings of burden to relatives from the perspective of care-recipients [29, 30]. Furthermore, most of these studies are restricted to palliative care settings [31, 32]. Thus, future research is required to clarify this relationship.

The association between preferences for care in relatives’ homes and living with partner or spouse might be explained by the fact that compared to individuals living alone, individuals living with partner/spouse might have more family members or relatives who are willing to give aid or assistance. Thus, the living situation might be associated with the (perceived) availability of informal caregivers [6, 33].

We found that current need of care was significantly associated with long-term care preferences for home care and care in assisted living. This is also supported by several other studies in Germany and America [5–7, 34]. The current study also found that lower self-rated health was positively associated with preferences for care in nursing home/old age home and preferences for home care. These findings might be explained by the fact that individuals with low self-rated health prefer traditional care settings where they might receive strong support. However, this should be investigated in future studies. The relation between self-rated health and preferences for care in nursing home/old age home supports previous findings [4–7].

In total, need factors (self-rated health and morbidity) are strongly associated with preferences for care in assisted living as well as preferences for care in nursing home/old age home. This might be explained by the fact that individuals in need for care cannot be cared for outside an institutional setting to meet their basic needs (for example, bathing or using the toilet)—and satisfying the basic needs is of high importance to these individuals [35].

In our study, preferences for care in assisted living were positively associated with higher education. While this is in line with a previous study [6], overall the evidence is mixed [5, 34, 36]. For example, a study among older Korean Americans found that a higher educational level was associated with a lower probability of turning to all formal instead of all informal care settings. These differences might be mainly explained by discrepancies in cultural settings which in turn are related to expectations and family norms [4].

As for long-term care abroad, only a few studies investigated the predictors of preferences for care in a foreign country. We found that these preferences were positively associated with lower age and being born abroad. The former relation might be explained by the fact that individuals in higher age groups might have different values or traits (for example, openness to experience) compared with younger individuals. Moreover, younger individuals might have good knowledge of foreign languages which is important since, for example, many caregivers in Thailand do not speak German at all or speak German poorly [37]. This finding is also in accordance with a population-based survey of the German population aged 14 and above [17]. Furthermore, age is positively associated with knowledge of care [22]. Thus, younger individuals might have unrealistic expectations about his or her functional status in old age. The latter relation (preferences for care abroad and being born abroad) might be explained by the fact that individuals who report being born abroad might be more flexible and are likely to be more open to new experiences [38] since they have left their homeland at least once. Furthermore, this latter relationship might be explained by the fact that these individuals return to their countries of origin in order to be cared for and supported by their relatives. We assume that our findings are in line with a previous study which found that preferences for long-term care abroad was higher in urban population compared with rural population [17]. This might support the idea that country of origin and the degree of urbanization reflect unobserved factors such as openness to experience or flexibility [39].

It should be highlighted that our data were obtained from a large, population-based sample in individuals aged 65 and above. Furthermore, several care settings and numerous predictors were analyzed. Furthermore, this is one of the first studies examining the predictors of preferences for long-term care abroad. Four point (no mid-point) Likert scales were used. Thus, the chance to express a truly neutral position was not offered. However, the use of the four point scales might help to alleviate social desirability bias because it might change the intensity of the preferences [40].

As this is a cross-sectional study, it is difficult to determine whether the statistical association identified reflect causal relations. Thus, longitudinal studies are needed. Longitudinal studies are also needed to guide policy makers. Moreover, we assume that other unobserved factors such as personality traits (e.g. neuroticism, extraversion etc.) might play a role in long-term care preferences [41]. Furthermore, our instruments should be validated in future studies. Individuals were asked to report their preferences for care settings (at own home; in relatives’ homes; in assisted living; in nursing home/old age home; in a foreign country). However, we cannot conclude which care setting is preferred most (without making further assumptions). Thus, due to this fact and due to the cross-sectional nature of our study policy implications are limited.

## Conclusions

Numerous variables used are occasionally significant, underlining the complex nature of long-term care preferences. A better understanding of factors which are associated with preferences for care settings might contribute to improving long-term care health services. This might help to improve the satisfaction of care-recipients with long-term care services.

## Additional file

Additional file 1: Data. Data set. (CSV 269 kb)

## Abbreviations

ADM: Arbeitskreis Deutscher Markt- und Sozialforschungsinstitute
CATI: Computer Assisted Telephone Interview
OR: Odds ratio
USUMA: Unabhängige Serviceeinrichtung für Umfragen, Methoden und Analysen

## References

[1] Kinsella K, Wan H (2009). An aging world 2008.

[2] Matthews Z, Channon A, Van Lerberghe W (2006). Will there be enough people to care? Notes on workforce implications of demographic change 2005–2050.

[3] Heuchert M, König H-H, Lehnert T. Die Rolle von Präferenzen für Langzeitpflege in der sozialen Pflegeversicherung–Ergebnisse von Experteninterviews. Gesundheitswesen. 2016;1–6. doi:10.1055/s-0041-111839. 10.1055/s-0041-111839 26990612

[4] McCormick WC, Ohata CY, Uomoto J, Young HM, Graves AB, Kukull W, Teri L, Vitaliano P, Mortimer JA, McCurry SM (2002). Similarities and differences in attitudes toward long‐term care between Japanese Americans and Caucasian Americans. J Am Geriatr Soc.

[5] Min JW (2005). Preference for long-term care arrangement and its correlates for older Korean Americans. J Aging Health.

[6] Pinquart M, Sörensen S (2002). Older adults’ preferences for informal, formal, and mixed support for future care needs: a comparison of Germany and the United States. Int J Aging Hum Dev.

[7] Pinquart M, Sörensen S, Davey A (2003). National and regional differences in preparation for future care needs: A comparison of the United States and Germany. J Cross Cult Gerontol.

[8] Hajek A, Brettschneider C, Ernst A, Posselt T, Wiese B, Prokein J, Weyerer S, Werle J, Fuchs A, Pentzek M (2016). Longitudinal predictors of informal and formal caregiving time in community-dwelling dementia patients. Soc Psychiatry Psychiatr Epidemiol.

[9] Statistisches Bundesamt (2009). Bevölkerung Deutschlands bis 2060–12. koordinierte Bevölkerungsvorausberechnung.

[10] Statistisches Bundesamt (2009). Bevölkerung und Erwerbstätigkeit. Entwicklung der Privathaushalte bis 2030. Ergebnisse der Haushaltsvorausberechnung.

[11] Au C, Sowarka D (2007). Die Vereinbarkeit von Pflege und Erwerbstätigkeit. Informationsdienst Altersfragen.

[12] Hajek A, König H-H (2016). The effect of intra-and intergenerational caregiving on subjective well-being–evidence of a population based longitudinal study among older adults in Germany. PLoS ONE.

[13] Hajek A, König H-H (2016). Informal caregiving and subjective well-being: evidence of a population-based longitudinal study of older adults in Germany. J Am Med Dir Assoc.

[14] Haley WE (2003). Family caregivers of elderly patients with cancer: understanding and minimizing the burden of care. J Support Oncol.

[15] Cvengros JA, Christensen AJ, Cunningham C, Hillis SL, Kaboli PJ. Patient preference for and reports of provider behavior: impact of symmetry on patient outcomes. Health Psychol. 2009;28(6):660-7. 10.1037/a0016087 19916633

[16] Parley FF (2001). Person-centred outcomes Are outcomes improved where a person-centred care model is used?. J Intellect Disabil.

[17] KONPRESS-Medien eG. Pflege im Ausland – Im Alter ins Exil? 2013.

[18] Ormond M, Toyota M. Confronting economic precariousness through international retirement migration: Japan’s old-age ‘economic refugees’ and Germany’s ‘exported grannies’. In: Rickly J, Hannam K, Mostafanezhad M, editors. Tourism and leisure mobilities: politics, work and play. Abingdon: Routledge; 2016. p. 134–146.

[19] Toyota M (2006). Ageing and transnational householding: Japanese retirees in Southeast Asia. Int Dev Plan Rev.

[20] Hajek A, Brettschneider C, Ernst A, Posselt T, Mamone S, Wiese B, Weyerer S, Werle W, Pentzek M, Fuchs A, Stein J, Luck T, Bickel H, Mösch E, Heser K, Kleineidam L, Maier W, Scherer M, Riedel-Heller S, HH K. Einflussfaktoren auf die Pflegebedürftigkeit im Längsschnitt. Gesundheitswesen. 2016;1–6. doi:10.1055/s-0041-111841. 10.1055/s-0041-111841 27056709

[21] Hajek A, Brettschneider C, Lange C, Posselt T, Wiese B, Steinmann S, Weyerer S, Werle J, Pentzek M, Fuchs A, Stein J, Luck T, Bickel H, Mösch E, Wagner M, Jessen F, Maier W, Scherer M, Riedel-Heller S, König H (2015). Longitudinal predictors of institutionalization in old age. PLoS ONE.

[22] Kuhlmey A, Suhr R, Blüher S, Dräger D. Das Risiko der Pflegebedürftigkeit. Pflegeerfahrungen und Vorsorgeverhalten bei Frauen und Männern zwischen dem 18. und 79. Lebensjahr. In Böcken J, Braun B, Repschläger Uwe (Eds.): Gesundheitsmonitor 2013. Bürgerorientierung im Gesundheitswesen. Kooperationsprojekt der Bertelsmann Stiftung und der BARMER GEK (S. 11-38). Gütersloh: Bertelsmann Stiftung. 2013.

[23] Hajek A, Lehnert T, Wegener A, Riedel-Heller SG, König H-H. Informelles Pflegepotenzial bei Älteren in Deutschland. Z Gerontol Geriatr. 2017;1–7. doi:10.1007/s00391-017-1181-y. 10.1007/s00391-017-1181-y 28127636

[24] Hajek A, Lehnert T, Wegener A, Riedel-Heller SG, König H-H. Langzeitpflegepräferenzen der Älteren in Deutschland – Ergebnisse einer bevölkerungsrepräsentativen Umfrage. Gesundheitswesen. in press. 10.1055/s-0042-124663 28268234

[25] Spangenberg L, Glaesmer H, Brähler E, Strauß B (2012). Use of family resources in future need of care. Care preferences and expected willingness of providing care among relatives: a population-based study. Bundesgesundhbl Gesundheitsforsch Gesundheitsschutz.

[26] McAuley WJ, Blieszner R (1985). Selection of long-term care arrangements by older community residents. Gerontologist.

[27] Kasper J, Shore A, Penninx B (2000). Caregiving arrangements of older disabled women, caregiving preferences, and views on adequacy of care. Aging Clin Exp Res.

[28] Eckert JK, Morgan LA, Swamy N (2004). Preferences for receipt of care among community-dwelling adults. J Aging Soc Policy.

[29] Löfqvist C, Granbom M, Himmelsbach I, Iwarsson S, Oswald F, Haak M (2013). Voices on relocation and aging in place in very old age—a complex and ambivalent matter. Gerontologist.

[30] Svidén G, Wikström B-M, Hjortsjö-Norberg M (2002). Elderly persons’ reflections on relocating to living at sheltered housing. Scand J Occup Ther.

[31] Givens JL, Mitchell SL (2009). Concerns about end-of-life care and support for euthanasia. J Pain Symptom Manage.

[32] Karlsson M, Strang P, Milberg A (2007). Attitudes toward euthanasia among Swedish medical students. Palliat Med.

[33] Bradley EH, Curry LA, Mcgraw SA, Webster TR, Kasl SV, Andersen R (2004). Intended use of informal long‐term care: the role of race and ethnicity. Ethn Health.

[34] Wolff JL, Kasper JD, Shore AD (2008). Long-term care preferences among older adults: a moving target?. J Aging Soc Policy.

[35] Harrefors C, Sävenstedt S, Axelsson K (2009). Elderly people’s perceptions of how they want to be cared for: an interview study with healthy elderly couples in Northern Sweden. Scand J Caring Sci.

[36] Wielink G, Huijsman R, McDonnell J (1997). Preferences for care a study of the elders living independently in the Netherlands. Res Aging.

[37] Horn V, Schweppe C (2016). Transnational aging: current insights and future challenges.

[38] Canache D, Hayes M, Mondak JJ, Wals SC (2013). Openness, extraversion and the intention to emigrate. J Res Pers.

[39] McCann SJH (2011). Emotional health and the Big Five personality factors at the American state level. J Happiness Stud.

[40] Garland R (1991). The mid-point on a rating scale: Is it desirable. Mark Bull.

[41] Sörensen S, Duberstein PR, Chapman B, Lyness JM, Pinquart M (2008). How are personality traits related to preparation for future care needs in older adults?. J Gerontol B Psychol Sci Soc Sci.

